# Assessing the Global Impact of Brain Small Vessel Disease on Cognition: The Multi‐Ethnic Study of Atherosclerosis

**DOI:** 10.1002/alz.70326

**Published:** 2025-06-04

**Authors:** Sokratis Charisis, Tanweer Rashid, Christina Dintica, Mitzi Gonzales, Hangfan Liu, Jeffrey B. Ware, Thomas R. Austin, Paul N. Jensen, Alison E. Fohner, Jordan E. Tanley, Jingzhong Ding, José A. Luchsinger, Bonnie Sachs, Ilya M. Nasrallah, R. Nick Bryan, Kathleen M. Hayden, David Wolk, Katya Rascovsky, Christos Davatzikos, William T. Longstreth, Kristine Yaffe, Sudha Seshadri, Susan R. Heckbert, Timothy Hughes, Mohamad Habes

**Affiliations:** ^1^ Neuroimage Analytics Laboratory (NAL) and the Biggs Institute Neuroimaging Core (BINC) Glenn Biggs Institute for Alzheimer's and Neurodegenerative Diseases University of Texas Health Science Center San Antonio San Antonio Texas USA; ^2^ Department of Neurology University of Texas Health Science Center at San Antonio San Antonio Texas USA; ^3^ Department of Psychiatry and Behavioral Sciences University of California, San Francisco San Francisco California USA; ^4^ Department of Neurology Cedars Sinai Medical Center Los Angeles California USA; ^5^ Center for Advanced Imaging Research (CAIR) University of Maryland School of Medicine Baltimore Maryland USA; ^6^ Department of Radiology Perelman School of Medicine University of Pennsylvania Philadelphia Pennsylvania USA; ^7^ Department of Epidemiology University of Washington Seattle Washington USA; ^8^ Cardiovascular Health Research Unit University of Washington Seattle Washington USA; ^9^ Department of Medicine University of Washington Seattle Washington USA; ^10^ Department of Internal Medicine Wake Forest School of Medicine Winston‐Salem North Carolina USA; ^11^ Departments of Medicine and Epidemiology Columbia University Medical Center New York New York USA; ^12^ Department of Neurology Wake Forest University School of Medicine Winston‐Salem North Carolina USA; ^13^ AI2D Center for AI and Data Science for Integrated Diagnostics and Center for Biomedical Image Computing and Analytics University of Pennsylvania Philadelphia Pennsylvania USA; ^14^ Department of Social Sciences and Health Policy Division of Public Health Sciences Wake Forest University School of Medicine Winston‐Salem North Carolina USA; ^15^ Alzheimer's Disease Research Center University of Pennsylvania Philadelphia Pennsylvania USA; ^16^ Department of Neurology University of Pennsylvania Philadelphia Pennsylvania USA; ^17^ Department of Neurology University of Washington Seattle Washington USA

**Keywords:** aging, atrophy, attention, cardiovascular disease risk, cerebral small vessel disease, cognition, cognitive performance, deep learning, delayed memory, diffusion tensor imaging, enlarged perivascular spaces, executive function, factor analysis, fractional anisotropy, Framingham risk score, global cognition, gray matter volume, immediate memory, language, latent variable, magnetic resonance imaging, mediation, microbleeds, phonemic fluency, processing speed, semantic fluency, structural equation modeling, trace, visuospatial functioning, white matter hyperintensities

## Abstract

**INTRODUCTION:**

We aimed to examine the global impact of brain small vessel disease (SVD) on cognitive performance.

**METHODS:**

In 892 participants from the Multi‐Ethnic Study of Atherosclerosis (MESA), we derived perivascular spaces (PVS), white matter hyperintensities (WMH), microbleeds (MB), and white matter fractional anisotropy (FA) and trace (TR). Cognitive function was assessed with a comprehensive neuropsychological battery.

**RESULTS:**

A composite SVD measure was constructed as a linear combination of basal ganglia PVS, thalamus PVS, periventricular WMH, subcortical WMH, and white matter FA and TR, and exhibited associations with worse global and domain‐specific cognitive performance. Additionally, SVD mediated the effect of age and cardiovascular disease risk on global cognitive function, both directly and through smaller gray matter (GM) volume.

**DISCUSSION:**

Integrating multiple individual SVD endophenotypes may more accurately reflect the neurobiology of SVD and capture its global impact on cognition. SVD mediates the effects of age and cardiovascular disease risk on cognition through both atrophy‐related and non–atrophy‐related pathways.

**Highlights:**

- Associations between individual magnetic resonance imaging (MRI) markers of brain small vessel disease and cognitive outcomes might not fully capture the global impact of small vessel disease on cognition.
- We modeled small vessel disease as a latent construct, integrating multiple MRI endophenotypes in strategic brain regions.
- The small vessel disease construct was associated with worse global and domain‐specific cognitive performance.
- The small vessel disease construct exhibited mediating effects in the relationships of aging and cardiovascular disease risk with cognition through pathways that both involve and are independent of brain atrophy.
- Integrating information from multiple relevant imaging endophenotypes could open new avenues in small vessel disease research, broadening our understanding of its risk factors and clinical correlates.

## 1 BACKGROUND

Cerebral small vessel disease (SVD) is thought to account for up to 25% of ischemic strokes worldwide and is the most important vascular contributor to cognitive decline and dementia.^1^ This makes its accurate and timely characterization with the available diagnostic means (e.g., neuroimaging coupled with advanced deep learning) an important public health priority.

With the development and advancement of brain magnetic resonance imaging (MRI), an expanding spectrum of tissue alterations considered to represent different endophenotypes of SVD pathology has been identified. These tissue alterations, among others, include white matter hyperintensities (WMH), perivascular spaces (PVS), microbleeds (MB), and, more recently, markers of white matter (WM) microstructural damage.^1^, ^2^ Research exploring the relationships of SVD with cognition has primarily focused on studying associations with individual MRI measures, such as total WMH,^3^, ^4^ PVS,^5^, ^6^ and MB^7^, ^8^ burden. However, brain aging and pathologies other than SVD, such as Alzheimer's disease or cerebral amyloid angiopathy, can give rise to similar findings on brain MRI,^9^, ^10^, ^11^, ^12^, ^13^ making it difficult to distinguish the extent to which observed associations can be attributed to SVD versus other factors such as aging or neurodegeneration. Moreover, individual SVD endophenotypes may only reflect specific components of the disease rather than capturing the global impact of SVD on cognitive outcomes.^14^ Therefore, the development of methods that effectively combine the information conveyed by different SVD endophenotypes to accurately and comprehensively examine the associations of SVD with risk factors and health outcomes of interest remains an important challenge that has yet to be addressed.

The spatial distribution of SVD endophenotypes within the brain may help determine their underlying pathogenesis.^9^, ^10^ For example, previous research has shown that periventricular and subcortical WMH reflect distinct pathological changes,^11^ PVS in different brain regions may have different pathophysiologies,^15^, ^16^ and lobar and deep MB are differentially associated with vascular and amyloid‐related pathologies.^17^, ^18^ Therefore, the location of these MRI markers may encode crucial information that could help identify patterns with similar etiopathogenetic substrates. However, leveraging this information in large‐scale epidemiological data had, until recently, been particularly challenging, due to the impracticality and labor intensity of the manual methods used for SVD marker detection and quantification.^16^, ^17^

In this study, by leveraging advanced deep learning algorithms, we derived previously proposed MRI markers of SVD in different brain regions and computed WM microstructural integrity indices in a multi‐ethnic community‐based sample. We explored the latent structure of these measures to identify spatial patterns most consistent with SVD pathology and constructed a composite SVD measure that incorporates information from multiple relevant endophenotypes. We then examined associations of this composite measure with global and domain‐specific cognitive performance, and tested for potential mediating effects of SVD in the relationships of age and cardiovascular disease risk with cognition.

## 2 METHODS

### 2.1 Participants

The Multi‐Ethnic Study of Atherosclerosis (MESA) is a longitudinal study designed to investigate subclinical cardiovascular disease in a diverse community‐based sample of 6814 men and women aged 45–84 years at enrollment. Participants were recruited from six US‐based sites (Baltimore City and Baltimore County, Maryland; Chicago, Illinois; Forsyth County, North Carolina; Los Angeles County, California; New York, New York; and St. Paul, Minnesota).^19^ At MESA exam 6, 1062 participants underwent brain MRI from 2018 to 2019 in the setting of the Atrial Fibrillation ancillary study^20^ (Methods S1); of these, 892 participants also underwent a complete neuropsychological evaluation as part of one of the following ancillary studies: the Epigenetics of Cognitive Function (from 2016 to 2017, *n* = 76) or MESA Memory (from 2018 to 2019, *n* = 115) at MESA exam 6, or MIND visit A (from 2019 to 2022, *n* = 701) (Figure S1). Each study site obtained institutional review board approval, and all participants provided written informed consent.

### 2.2 Brain MRI and SVD marker measurements

The detailed MESA brain MRI protocol has been described elsewhere.^21^ Briefly, scans were acquired using 3 Tesla Siemens scanners. Structural sequences included 1 mm isotropic sagittal 3D T1‐weighted, T2‐weighted, and fluid‐attenuated inversion recovery (FLAIR) sequences, susceptibility weighted imaging (SWI)/quantitative susceptibility mapping (QSM), and axial 2D, 30‐direction echo‐planar diffusion‐tensor imaging (DTI). An automated preprocessing pipeline including inhomogeneity correction^22^ and extraction of the intracranial brain tissues and cerebrospinal fluid using multi‐atlas skull‐stripping^23^ was applied. Anatomical regions of interest (ROIs) were identified using a multi‐atlas label fusion method to segment gray matter (GM) and white mWM volume (in mm^3^).^24^ The total intracranial volume (ICV) was calculated as the sum of GM, WM, and cerebrospinal fluid volumes.

**RESEARCH IN CONTEXT**

1. **Systematic review**: The literature was reviewed using traditional (e.g., PubMed) sources. Research exploring the relationships between brain small vessel disease and cognition has primarily focused on associations with individual magnetic resonance imaging (MRI) markers. These individual markers might only reflect certain aspects of small vessel disease, potentially failing to capture its global impact on cognitive function.
2. **Interpretation**: Our findings suggest that, instead of relying on individual MRI markers, integrating information from multiple small vessel disease endophenotypes might more accurately capture its associations with, and mediating effects on, cognitive outcomes.
3. **Future directions**: This work introduces analytical frameworks that model small vessel disease by integrating multiple imaging endophenotypes in strategic brain regions. Such frameworks could be utilized by future studies to better reflect the neurobiology of small vessel disease than individual MRI markers, thereby enhancing our understanding of its risk factors and its impact on cognitive and other health outcomes.

#### 2.2.1 WMH detection and mapping

The volume of WMH was measured from inhomogeneity‐corrected and co‐registered FLAIR and T1‐weighted images using a deep learning‐based method.^25^ WMH were grouped into periventricular and subcortical, as described elsewhere.^26^, ^27^

#### 2.2.2 PVS detection and mapping

Quantification of PVS counts was performed from T2‐weighted, T1‐weighted, and FLAIR images using a deep learning‐based model (Methods S2).^16^, ^28^ Similarly to our previously described approach, PVS were grouped into six anatomic regions: basal ganglia, thalamus, insular, brainstem, frontoparietal, and temporal.^16^

#### 2.2.3 MB detection and mapping

Quantification of MB counts was performed from T2‐weighted images and SWI/QSM using a deep learning‐based model (Methods S2).^29^ MB were grouped into lobar, deep, and infratentorial; as previously described.^17^

#### 2.2.4 Measures of WM microstructural integrity

Compared to conventional MRI measures, DTI holds the distinct advantage of capturing subtle microstructural changes in normal‐appearing WM, and it is currently recognized as a marker of SVD.^2^ We, therefore, included in our analysis DTI measures that are particularly sensitive to WM microstructural damage.^2^ Specifically, we calculated the mean WM fractional anisotropy (FA, expressing the degree to which a single diffusion orientation is dominant) and trace (TR, expressing the total apparent diffusion as the sum of the diagonal elements of the diffusion tensor) using automated DTI‐processing pipelines.^30^

### 2.3 Cognitive assessment

Cognitive function was assessed in four languages (English, Spanish, Mandarin, and Cantonese – based on participant preference) with a neuropsychological test battery consisting of the following tests: the Cognitive Abilities Screening Instrument (CASI, version 2),^31^ the Digit Symbol Coding test,^32^ the Digit Span test forwards and backwards,^32^ the National Alzheimer's Coordinating Center Uniform Data Set version 3 (UDS v3) neuropsychological battery,^33^ the Wide Range Achievement Test 5 (WRAT 5),^34^ the Auditory‐Verbal Learning Test (AVLT),^35^ and the Quick Dementia Rating System (QDRS).^36^ Study participants were also asked to identify an informant who can best answer questions about their daily life. The informant completed the QDRS, the Neuropsychiatric Inventory Questionnaire (NPI‐Q),^37^ and the Functional Activities Questionnaire (FAQ/FAS).^38^ Individual test scores were normalized, using either published (for UDSv3^39^, ^40^ and AVLT^41^) or self‐norms (for Chinese American participants in English and Chinese languages), and were combined based on a priori neuropsychological knowledge of particular cognitive functions that each test primarily examines,^33^ to produce average domain scores for immediate memory, delayed memory, language/semantic fluency, phonemic fluency, attention/processing speed, executive, and visuospatial functioning (Table S1). Subsequently, a principal component analysis including all normalized scores from cognitively normal participants was performed, and the first principal component loadings were used as weights to calculate a global cognitive score as a weighted sum of all individual test scores. Higher global and domain‐specific scores indicate better cognitive performance. For participants enrolled in more than one ancillary study, the cognitive assessment that was chronologically closest to brain MRI was used.

### 2.4 Demographics, vascular risk factors, and other covariates

Self‐reported age, sex, race/ethnicity, and maximum attained level of education were recorded at the baseline MESA exam (from 2000 to 2002). At MESA exam 6 (from 2016 to 2018), vascular risk factor data were collected, information on smoking status, alcohol consumption, medication use, and intentional physical activity (in metabolic equivalents of task – MET/minute/week) were updated, and anthropometric data were measured. Waist‐to‐hip ratio (WHR) was calculated by dividing the participant's waist circumference (in cm) by hip circumference (in cm). Systolic blood pressure (SBP, in mmHg) was calculated as the average of the last two out of three measurements taken with the participant resting in a sitting position. Hyperlipidemia was defined as reported use of lipid‐lowering medications. Diabetes mellitus was defined as reported use of glucose‐lowering medications, fasting blood glucose ≥126 mg/dL, or hemoglobin A1c ≥6.5%. The Framingham risk score for global cardiovascular disease (FRS) was calculated based on exam 6 values.^42^ Apolipoprotein E (*APOE)* ε4 allele carriership was determined from single nucleotide polymorphisms (rs429358 and rs7412).

### 2.5 Statistical analysis

Continuous variable distributions were graphically explored using Q‐Q plots and Kernel density plots. Variables expressing regional MB counts were dichotomized (absence/presence). Continuous MRI marker variables were converted to *z*‐scores to unify their scales. The variable expressing intentional physical activity underwent Tukey's “ladder of powers” transformation to normalize its distribution.^43^

Participant characteristics were compared using analysis of variance, Mann–Whitney–Wilcoxon tests, and Pearson's chi‐squared, as appropriate.

#### 2.5.1 MRI marker dimensions and associations with cognitive performance

To investigate the number and nature of latent constructs represented by the different MRI markers, we conducted an exploratory factor analysis (EFA). To incorporate dichotomous variables (indicating the presence of MB), we constructed a mixed correlation matrix containing Pearson (between continuous variables), tetrachoric (between dichotomous variables), and biserial (between continuous and dichotomous variables) correlations. The suitability of correlation matrix for EFA was assessed with the Kaiser–Meyer–Olkin (KMO) test. Considering that matrices containing polychoric correlations may be non‐positive definite,^44^ a factoring method that does not require matrix inversion and with good performance on polychoric correlation matrices (minimum residual [minres]) was used.^45^ The number of latent factors to be extracted was determined by the very simple structure (VSS) and minimum average partial (MAP) criteria.^46^, ^47^ Indicators with communalities (h^2^) <0.2 were removed using an iterative process (removing the item with the lowest communality and repeating the analysis until all item communalities were ≥0.2).^48^ Factor axes were initially rotated using an oblique rotation (oblimin) to test for potential correlations among factors; for correlations <0.32 (<10% overlap in variance), factors were assumed to be orthogonal and the rotation was changed to varimax.^49^ Rotated factor loadings of >0.32 (>10% of indicator variance explained by the factor) were analyzed to determine the nature of latent construct represented by each factor.^49^

As an exploratory step, we also conducted an extension analysis to determine the position of the excluded variables within the factor space.^50^

Factor scores were estimated using the regression method,^51^ and their associations with global and domain‐specific cognitive performance were assessed with generalized linear models (Gaussian family, identity link function). In these models, the cognitive scores were the outcomes, and the factor scores were the main predictors (in separate models for each factor). Confounder adjustment was conducted in three steps: (i) Model 1 was adjusted for age, sex, race/ethnicity, education, study site, ICV, language of cognitive testing, interval between MRI scan and cognitive assessment, and order of MRI scan and cognitive assessment completion; (ii) Model 2 was further adjusted for vascular risk factors (systolic blood pressure, use of antihypertensive medications, diabetes, hyperlipidemia, current smoking, current alcohol consumption, WHR, and intentional physical activity); and (iii) Model 3 was further adjusted for *APOE* ε4 carrier status. Profile‐likelihood‐based 95% confidence intervals (CIs) were computed for all models.^52^

#### 2.5.2 Structural equation modeling

We examined the potential mediating effects of SVD on cognitive aging using the structural equation modeling (SEM) framework.^53^ The measurement model modeled SVD as a latent variable with variables loaded onto the factor assumed to represent SVD as indicators. Residual terms of indicators expressing the same MRI marker in different brain regions were allowed to covary to account for shared method variance.^54^ The structural model modeled the global cognitive score as the outcome, age as the independent variable, and SVD as a mediator. Based on prior knowledge, we refined the model specification by adding a pathway from the independent variable to cognition through GM volume, considering that brain atrophy has been associated with cognitive decline both in the setting of aging as well as cardiovascular disease,^55^, ^56^ and a pathway from SVD to GM volume, considering prior evidence that SVD could lead to brain atrophy.^1^, ^57^, ^58^

Given previously reported associations of higher cardiovascular disease risk with worse cognitive performance,^59^ we constructed a similarly structured model after substituting age with FRS^42^ to study the potential mediating effects of SVD in the relationship of cardiovascular disease risk with cognition.

These analyses were adjusted for basic demographics (sex, race/ethnicity, education), study site, and ICV. Models with FRS as the independent variable were not adjusted for sex, since functions for FRS calculation are sex‐specific.^42^ Parameter estimates were computed using the maximum likelihood estimator, and bias‐corrected 95% CIs for indirect and direct effects were calculated using 5000 bootstrap samples.^60^

To determine whether an advantage exists in modeling SVD as a latent construct instead of relying on single MRI measures, we recomputed the structural part of the SEM models while substituting the latent SVD variable with each of its individual indicators.

Lastly, to assess the robustness of the SVD latent construct to potential inter‐site or inter‐scanner differences, we implemented a multi‐group confirmatory factor analysis (MGCFA) invariance strategy to (i) evaluate whether the relationships between the SVD construct and its MRI indicators were consistent across different study sites/scanners, and (ii) assess the inter‐site/scanner reliability of the MRI indicators (Methods S3).

All SEM models were computed using the lavaan package.^61^

#### 2.5.3 Supplementary analyses

To further demonstrate the multidimensionality of MRI markers but also facilitate the comparability of our findings to prior studies relying on individual SVD endophenotypes, in supplementary analyses we examined the associations of individual MRI markers with global and domain‐specific cognitive performance. In these analyses, variables expressing regional WMH volumes underwent Tukey's “ladder of powers” transformation to normalize their distributions.^43^ Models were similarly structured to those described in section 2.5.1. To account for testing multiplicity, the false discovery rate (FDR) within each cognitive domain was controlled at <5% using the Benjamini–Hochberg procedure.^62^

Analyses and graphics were performed using R, version 4.3.1 (R Foundation for Statistical Computing, 2022). All statistical tests were unpaired. A two‐sided *p*‐value of <0.05 was considered statistically significant.

### Data availability

Deidentified data not published within this article can be made available upon request at https://www.mesa‐nhlbi.org/. The source code for the conducted analyses is available at https://github.com/UTHSCSA‐NAL/SVD_Cognition_MESA.

## 3 RESULTS

### 3.1 Missing data analysis

A total of 1041 participants had complete MRI marker data (Figure S1); of those, cognitive data were available for 892. Participants with available cognitive data were younger, more physically active, and had a different racial/ethnic composition (more White and Black participants, fewer Chinese American and Hispanic participants), higher education, lower prevalence of diabetes, higher prevalence of current alcohol consumption, slightly lower WHR, and lower FRS, compared to those without (*n* = 149, Table S2). Regarding MRI measures, those with available cognitive data had higher GM volume, less basal ganglia PVS, higher FA, and lower TR, compared to those without.

### 3.2 Participant characteristics

Participant characteristics (*N* = 892) are presented in Table 1. Mean (SD) age at brain MRI was 73.6 (7.9) years; 477 (53%) participants were women. Participants were of White (*n* = 379 [42%]), Black (237 [27%]), Hispanic (157 [18%]), and Chinese American (119 [13%]) race/ethnicity. Regarding vascular risk factors, 509 (57%) participants reported use of antihypertensive medications, 182 (21%) had diabetes, and 400 (45%) had hyperlipidemia; mean WHR was 0.97 (0.06) for men and 0.90 (0.08) for women.

**TABLE 1:** Participant characteristics (*N* = 892)

*Demographic and vascular risk factors*	
Age, mean (SD), years	73.6 (7.9)
Sex, *n* (%)	
Women	477 (53%)
Men	415 (47%)
Race/ethnicity, *n* (%)	
White	379 (42%)
Chinese American	119 (13%)
Black	237 (27%)
Hispanic	157 (18%)
Education, *n* (%)	
Highschool or lower	213 (24%)
Less than bachelor's degree	272 (30%)
Bachelor's degree	187 (21%)
Graduate/professional school	220 (25%)
Systolic blood pressure, mean (SD), mmHg	126.6 (19.9)
	Missing (*n*): 2
Use of antihypertensives, n (%)	
No	378 (43%)
Yes	509 (57%)
Missing (n)	5
Diabetes mellitus, n (%)	
No	702 (79%)
Yes	182 (21%)
Missing (n)	8
Hyperlipidemia, n (%)	
No	487 (55%)
Yes	400 (45%)
Missing (n)	5
Current cigarette smoking, n (%)	
No	841 (94%)
Yes	51 (6%)
Current alcohol use, n (%)	
No	466 (52%)
Yes	426 (48%)
Waist‐to‐hip ratio, mean (SD), cm/cm	
Women	0.90 (0.08)
Men	0.97 (0.06)
Total intentional exercise, median (IQR), MET/min/week	1,102.5 (325.0, 2,460.0)
	Missing (n): 2
Framingham risk score for global cardiovascular disease, mean (SD)	17 (9)
	Missing (n): 8
*APOE* ε4 allele carrier, n (%)	
No	615 (73%)
Yes	233 (27%)
Missing (n)	44
*Brain MRI measures of interest*	
Intracranial volume, mean (SD), mm^3^	1,358,887 (145,867)
Gray matter volume, mean (SD), mm^3^	599,773 (64,803)
PVS, mean (SD), count	
Basal ganglia	63.3 (16)
Frontoparietal	403.9 (154.2)
Temporal	122.4 (60.4)
Insular	5.8 (5.1)
Brainstem	9.2 (3.6)
Thalamus	8.7 (5.1)
WMH volume, median (IQR), mm^3^	
Periventricular	2,351 (998, 6,143)
Subcortical	251 (43, 946)
Presence of microbleeds, n (%)	
Infratentorial	67 (8%)
Deep	121 (14%)
Lobar	216 (24%)
Mean white matter DTI measures	
Fractional anisotropy, mean (SD)	0.393 (0.025)
Trace, mean (SD)	0.0025 (0.0001)
*Cognitive measures*	
Language of cognitive testing, n (%)	
Cantonese	46 (5%)
English	768 (86%)
Mandarin	20 (2%)
Spanish	58 (7%)
Global composite cognitive score, mean (SD)	−0.72 (2.18)
Immediate memory domain, mean (SD)	−0.22 (0.89)
	Missing (*n*): 5
Delayed memory domain, mean (SD)	−0.18 (0.89)
	Missing (*n*): 6
Executive domain, mean (SD)	−0.29 (0.99)
	Missing (*n*): 1
Language/semantic fluency domain, mean (SD)	−0.10 (0.93)
	Missing (*n*): 8
Phonemic fluency domain, mean (SD)	−0.31 (0.98)
	Missing (*n*): 90
Attention/processing speed domain, mean (SD)	−0.15 (0.91)
Visuospatial domain, mean (SD), sdu	−0.38 (1.34)
	Missing (*n*): 225

Abbreviations: *APOE*, apolipoprotein E; DTI, diffusion tensor imaging; IQR, interquartile range; MET, metabolic equivalent of task; MRI, magnetic resonance imaging; PVS, perivascular spaces; SD, standard deviation; WMH, white matter hyperintensities.

For PVS, frontoparietal was the anatomic region with the highest count (mean, 403.9 [SD, 154.2]), followed by (in decreasing order) the temporal (122.4 [60.4]), basal ganglia (63.3 [16]), brainstem (9.2 [3.6]), thalamus (8.7 [5.1]), and insular (5.8 [5.1]) regions. WMH volume was higher in periventricular (median [IQR] = 2,351 [998, 6,143] mm^3^) and lower in subcortical (251 [43, 946] mm^3^) regions. MB were most prevalent in lobar regions (*n* = 216 [24%]), followed by the deep regions (121 [14%]) and the infratentorial regions (67 [8%]).

### 3.3 MRI marker dimensions

The MRI marker correlation matrix had an acceptable overall measure of sampling adequacy (0.71) based on the KMO test. Both the VSS and MAP criteria suggested extraction of two factors (Figure S2). Four indicators with communalities <0.2 were successively removed from the factor model (lobar MB [h^2^ = 0.07], brainstem PVS count [h^2^ = 0.09], infratentorial MB [h^2^ = 0.13], and deep MB [h^2^ = 0.19]). Oblique rotation revealed an inter‐factor correlation of 0.005; therefore, factors were assumed to be orthogonal, and the varimax solution was interpreted. Rotated factor loadings are reported in Table 2. Factor 1 explained 31% of the MRI marker variance and exhibited loadings for periventricular WMH (0.85), WM TR (0.84), WM FA (‐0.73), subcortical WMH (0.66), basal ganglia PVS (0.50), and thalamus PVS (0.42). Factor 2 explained 24% of the MRI marker variance and exhibited loadings for frontoparietal (0.94), temporal (0.90), and insular (0.57) PVS. No appreciable cross‐loadings were observed.

**TABLE 2:** Factor analysis of the MRI markers of small vessel disease

Parameter	Factor 1	Factor 2
Rotated factor loadings^a^		
Perivascular spaces		
Basal ganglia	**0.50** ^b^	0.14
Frontoparietal	0.03	**0.94** ^b^
Temporal	0.05	**0.90** ^b^
Insular	0.00	**0.57** ^b^
Thalamus	**0.42** ^b^	0.16
White matter hyperintensities		
Periventricular	**0.85** ^b^	−0.13
Subcortical	**0.66** ^b^	−0.14
Mean white matter DTI measures		
Fractional anisotropy	−**0.73** ^b^	0.01
Trace	**0.84** ^b^	0.07
Variance explained		
	0.31	0.24

*Note*: N = 892. The extraction method was minimum residual with an orthogonal (varimax) rotation.

Abbreviation: DTI, diffusion tensor imaging.

^a^ Values represent standardized factor loadings.

^b^ Factor loading of magnitude ≥0.32.

Extension analysis revealed that deep MB were related to Factor 1, whereas the rest of the excluded variables were not related to the extracted factors (Table S3).

#### 3.3.1 Interpretation of MRI marker dimensions

Extensive clinical and pathological evidence has linked WMH to SVD.^63^, ^64^ Furthermore, DTI measures have been suggested as early and sensitive markers of the microstructural WM damage that occurs in the setting of SVD.^2^, ^65^ Lastly, we have previously demonstrated in MESA participants that a high PVS burden in deep brain structures (i.e., basal ganglia and thalamus) is more likely to reflect underlying SVD than PVS burden in other brain regions.^16^ Based on these prior insights, Factor 1 was assumed to represent SVD. Factor 2 was, however, more difficult to interpret. Little is known about PVS in the frontoparietal, temporal, and insular regions. Some associations of frontoparietal PVS with vascular risk factors have been reported but have not been consistent across studies.^66^, ^67^ Therefore, Factor 2 was assumed to represent an unknown construct.

#### 3.3.2 MRI marker dimensions and cognitive performance

Associations of the extracted factors with cognition are presented in Table 3. Higher Factor 1 was associated with worse global, immediate memory, delayed memory, executive, language, phonemic fluency, and attention cognitive scores, whereas higher Factor 2 was associated with better global and immediate memory scores. After further adjustment for vascular risk factors and *APOE* genotype, the associations of Factor 1 with immediate memory and of Factor 2 with global cognition did not remain significant.

**TABLE 3:** Associations of the extracted factors with cognitive performance

	Model 1^a^		Model 2^b^		Model 3^c^	
*Cognitive domain*	Factor 1	Factor 2	Factor 1	Factor 2	Factor 1	Factor 2
Global						
β	−**0.458** ^d^	**0.151** ^d^	−**0.437** ^d^	0.12	−**0.425** ^d^	0.12
95% CI	−**0.622,** −**0.294**	**0.002, 0.300**	−**0.605,** −**0.269**	−0.029, 0.269	−**0.600,** −**0.249**	−0.032, 0.271
Immediate memory						
β	−**0.073** ^d^	**0.093** ^d^	−**0.075** ^d^	**0.071** ^d^	−0.064	**0.071** ^d^
95% CI	−**0.143,** −**0.002**	**0.030, 0.156**	−**0.147,** −**0.002**	**0.008, 0.134**	−0.139, 0.012	**0.006, 0.135**
Delayed memory						
β	−**0.158** ^d^	0.06	−**0.152** ^d^	0.052	−**0.142** ^d^	0.048
95% CI	−**0.227,** −**0.089**	−0.002, 0.123	−**0.223,** −**0.082**	−0.011, 0.114	−**0.215,** −**0.068**	−0.016, 0.111
Executive						
β	−**0.11** ^d^	0.067	**−0.093** ^d^	0.063	**−0.09** ^d^	0.073
95% CI	**−0.189, −0.031**	−0.004, 0.138	**−0.175, −0.011**	−0.009, 0.135	**−0.176, −0.005**	1.20e‐04, 0.146
Language/semantic fluency						
β	**−0.163** ^d^	0.015	**−0.164** ^d^	0.008	**−0.167** ^d^	3.56E‐05
95% CI	**−0.236, −0.090**	−0.051, 0.081	**−0.240, −0.088**	−0.059, 0.075	**−0.246, −0.088**	−0.068, 0.068
Phonemic fluency						
β	**−0.125** ^d^	0.053	**−0.118** ^d^	0.046	**−0.141** ^d^	0.049
95% CI	**−0.213, −0.038**	−0.023, 0.129	**−0.208, −0.027**	−0.031, 0.122	**−0.235, −0.046**	−0.029, 0.128
Attention/processing speed						
β	**−0.172** ^d^	0.006	**−0.158** ^d^	4.04E‐04	**−0.153** ^d^	0.004
95% CI	**−0.242, −0.102**	−0.057, 0.070	**−0.230, −0.085**	−0.064, 0.065	**−0.229, −0.078**	−0.061, 0.069
Visuospatial						
β	−0.089	0.064	−0.069	0.04	−0.106	0.047
95% CI	−0.213, 0.034	−0.045, 0.173	−0.196, 0.058	−0.070, 0.149	−0.239, 0.028	−0.066, 0.159

*Note*: Values represent beta coefficients from generalized linear models with cognitive scores as the outcomes and the respective factor score as the main predictor.

Abbreviation: APOE, apolipoprotein E; CI, confidence interval MRI, magnetic resonance imaging.

^a^ Adjusted for age, sex, race/ethnicity, education, study site, total intracranial volume, language of cognitive testing, interval between MRI scan and cognitive testing, and order of MRI scan and cognitive testing completion.

^b^ Further adjusted for vascular risk factors (systolic blood pressure, use of antihypertensive medications, diabetes, hyperlipidemia, current smoking, current alcohol consumption, waist‐to‐hip ratio, and intentional physical activity).

^c^ Further adjusted for *APOE* ε4 carrier status.

^d^ Significant at *p* ≤.05.

### 3.4 Structural equation modeling

**FIGURE 1:**
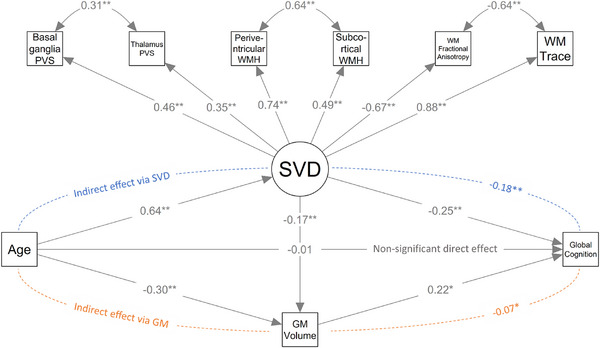
Path diagram of the conceptual model for the effect of age on cognition. In the measurement model, small vessel disease (SVD) is hypothesized as the underlying, or latent, cause of perivascular spaces (PVS) in the basal ganglia and thalamus, white matter hyperintensities (WMH) in periventricular and subcortical locations, and mean white matter (WM) fractional anisotropy and trace. In the structural model, SVD and gray matter (GM) volume are hypothesized as mediators of the effect of age on cognition. Arrow values in the measurement model represent standardized loadings (or residual covariances for double‐headed arrows), and in the structural model, comparative fit index standardized regression coefficients. Asterisks indicate statistically significant parameter estimates at *p* <0.01 (*) and *p* <0.001 (**). For calculation of the indirect effect via SVD, both the pathways Age→SVD→Cognition and Age→SVD→GMvolume→Cognition were considered.

In SEM models with age as the independent variable, higher age was associated with worse global cognition (total age effect: β [95% CI] = −0.071 [−0.089, −0.053]). There was evidence of mediation of the age effect by the specified indirect pathways, as indicated by the nonsignificant direct age effect (−0.003 [−0.028, 0.022]) and the significant total indirect effect (−0.068 [−0.089, −0.049]). The total indirect effect consisted of the following pathways: Age→SVD→Cognition (‐0.043 [‐0.061, ‐0.026]), Age→SVD→GMvolume→Cognition (−0.007 [−0.012, −0.003]), and Age→GMvolume→Cognition (−0.018 [−0.030, −0.007]). The path diagram with the completely standardized solution for this model is presented in Figure 1.

**FIGURE 2:**
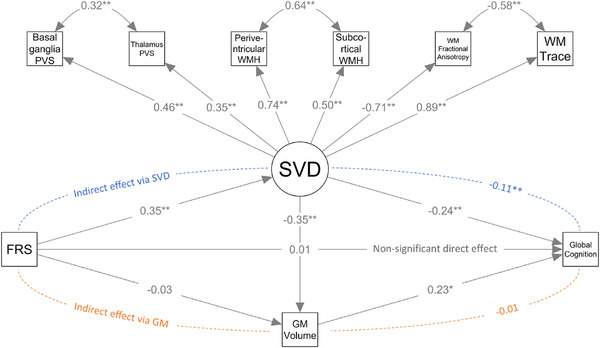
Path diagram of the conceptual model for the effect of cardiovascular disease risk on cognition. In the measurement model, small vessel disease (SVD) is hypothesized as the underlying, or latent, cause of perivascular spaces (PVS) in the basal ganglia and thalamus, white matter hyperintensities (WMH) in periventricular and subcortical locations, and mean white matter (WM) fractional anisotropy and trace. In the structural model, SVD and gray matter (GM) volume are hypothesized as mediators of the effect of Framingham cardiovascular disease Risk Score (FRS) on cognition. Arrow values in the measurement model represent standardized loadings (or residual covariances for double‐headed arrows), and in the structural model, standardized regression coefficients. Asterisks indicate statistically significant parameter estimates at *p* < 0.01 (*) and *p* < 0.001 (**). For calculation of the indirect effect via SVD, both the pathways FRS→SVD→Cognition and FRS→SVD→GMvolume→Cognition were considered.

In SEM models with FRS as the independent variable, higher FRS was associated with worse global cognition (total FRS effect: β [95% CI] = −0.028 [−0.044, −0.012]). There was evidence of mediation of the FRS effect by the specified indirect pathways, as indicated by the nonsignificant direct FRS effect (0.001 [−0.016, 0.018]) and the significant total indirect effect (−0.029 [−0.038, −0.021]). The total indirect effect consisted of the following pathways: FRS→SVD→Cognition (−0.021 [−0.030, −0.013]), FRS→SVD→GMvolume→Cognition (−0.007 [−0.012, −0.003]), and FRS→GMvolume→Cognition (−0.002 [−0.005, 0.000]). The path diagram with the completely standardized solution for this model is presented in Figure 2.

Model fit indices are reported in Table S4. Compared to individual MRI markers, SVD modeled as a latent construct mediated a greater portion of the effect of age and FRS on cognition (as evidenced by the larger indirect effects via SVD), and the respective models explained a higher proportion of the global cognitive score variance (Table S5).

The MGCFA model assuming only configural invariance (i.e., same SVD factor structure) across study sites demonstrated a good fit to the data [scaled χ^2^(df = 36) = 41.342, *p* = 0.25; comparative fit index [CFI] = 0.997; root mean square error of approximation [RMSEA] = 0.040; standardized root mean squared residual [SRMR] = 0.023], so we proceeded to test metric invariance by imposing equality constraints on the loadings of each MRI marker across sites. The metric invariance model also exhibited good fit [scaled χ^2^(df = 61) = 66.789, *p* = 0.29; CFI = 0.996; RMSEA = 0.035; SRMR = 0.063]. Subsequently, we compared the configural and metric invariance models using a likelihood ratio test with a scaled chi‐square difference test statistic.^68^ The test was not significant (*p* = 0.37), supporting metric invariance across different study sites. These findings indicate that the relationships between the SVD construct and its MRI indicators were consistent across sites (i.e., changes in the underlying SVD burden influenced the measured MRI markers to the same degree across different study sites).

Next, we further constrained the residual variances of each MRI marker to be the same across sites, and also the SVD latent factor variance to be equal across sites – thereby imposing equal MRI indicator reliability across sites. This model again demonstrated an acceptable fit [scaled χ^2^(df = 96) = 102.585, *p* = 0.3; CFI = 0.980; RMSEA = 0.060; SRMR = 0.213] that was not significantly worse than the metric invariance model (*p* = 0.1), supporting the assumption of equal MRI marker reliability across sites.

### 3.5 Supplementary analyses

Associations of individual MRI markers with global and domain‐specific cognitive performance (Model 1) are presented in Table S6. Higher basal ganglia PVS count was associated with worse global, language, and attention cognitive scores, while higher frontoparietal and insular region PVS counts were associated with better immediate memory scores. After adjustment for vascular risk factors (Model 2, Table S7) and *APOE* genotype (Model 3, Table S8), only the basal ganglia associations remained significant. There were no associations between cognitive scores and PVS count in other (i.e., temporal, brainstem, and thalamus) brain regions.

Higher periventricular WMH burden was associated with worse global, delayed memory, executive, language, phonemic fluency, and attention cognitive scores, while higher subcortical WMH burden was associated with worse global cognition. In Model 3, the associations of periventricular WMH with executive score and of subcortical WMH with global cognition did not remain significant.

MB presence in infratentorial, deep, or lobal regions was not associated with cognitive scores.

Higher WM FA was associated with better global, delayed memory, language, and attention cognitive scores, while higher WM TR was associated with worse global, delayed memory, executive, language, phonemic fluency, and attention scores. In Model 3, the association of WM TR with executive score did not remain significant.

## 4 DISCUSSION

In this investigation, we utilized advanced deep learning–based tools to derive previously proposed MRI markers of SVD in a multi‐ethnic community‐based sample. Given the spatial heterogeneity and multidimensionality of these markers, we analyzed their latent structure and constructed a single composite measure as a linear combination of the endophenotypes that best capture the SVD dimension. This composite measure was then applied to assess the global impact of SVD on cognitive performance. Finally, using SEM models, we demonstrated that SVD mediates the relationships of age and cardiovascular disease risk with cognition both through atrophy‐related and non–atrophy‐related pathways.

### 4.1 MRI marker dimensions and associations with cognitive performance

The interpretation of the MRI marker latent structure is described in section 3.4.1. Our results suggested that PVS in basal ganglia and thalamus measure underlying SVD. In line with these findings, we have previously found distinctive associations of PVS in the basal ganglia and thalamus with hypertension and other MRI markers of vascular brain injury in MESA, suggesting that PVS in these regions likely reflect underlying vascular pathology.^16^ Both WMH in periventricular as well as in subcortical locations loaded onto the factor representing SVD. Although periventricular WMH exhibit unique genetic architecture and histopathological characteristics compared to subcortical WMH,^11^, ^69^ it has been proposed that they are both part of the same pathological continuum, and more extensive WMH burden likely has a vascular etiology irrespective of its spatial pattern.^70^, ^71^ Finally, WM FA and TR loaded onto the SVD dimension, which is consistent with prior evidence that DTI measures are particularly sensitive to vascular WM damage, even in areas of normal‐appearing WM.^2^, ^65^ The extracted SVD factor exhibited associations with worse global cognition and poorer cognitive performance across all cognitive domains besides visuospatial functioning. These associations remained significant even after adjustment for vascular risk factors. Considering the spatial and pathogenetic heterogeneity of individual MRI markers, we aimed to extract a more specific SVD dimension and integrate various SVD endophenotypes in strategic locations into a single composite measure. This measure attempts to more accurately reflect the neurobiology of SVD and allows for studying associations of interest while bypassing multiple testing problems. Herein, we demonstrated the effectiveness of this approach in capturing associations with cognitive outcomes. However, the same approach could be utilized by future studies to explore relationships between SVD and other health‐related outcomes or risk factors of interest.

The pathophysiology underlying Factor 2 is unknown. We have previously found associations between greater temporal region PVS burden and larger total GM volume, and between greater insular region PVS burden and smaller total WMH volume in MESA,^16^ suggesting that PVS in these regions (and consequently Factor 2) might not necessarily represent underlying pathology but a (more) healthy aging dimension. The positive associations of Factor 2 with global cognition and immediate memory further suggest this possibility. Nevertheless, the etiology and potential clinical implications of PVS in these areas warrant further study.

Variables expressing brainstem PVS and MB presence were excluded from the final factor solution due to low communalities, indicating that they were not well explained by the factor model.^48^ Nonetheless, in extension analysis, deep MB appeared to be related to the SVD dimension, supporting the existing view that they reflect vascular brain injury.^18^

### 4.2 Mediating effects of SVD on cognitive performance

Compared to individual MRI markers, SVD modeled as a latent construct mediated the largest portion of the effect of age and cardiovascular disease risk on cognition. This underscores the ability of our latent variable approach to effectively detect mediation effects on exposure‐disease relationships by integrating information from multiple relevant SVD endophenotypes—each potentially capturing different disease aspects—into a single dimension, while also accounting for measurement error.

SEM analysis of the effect of age on cognition revealed significant indirect effects through SVD and brain atrophy pathways, mediating approximately 96% of the total age effect. Specifically, SVD through both atrophy‐related and non–atrophy‐related pathways mediated ∼71% of the total age effect, while atrophy pathways not related to SVD mediated ∼25% of the total age effect. In line with prior evidence suggesting that SVD might lead to brain atrophy,^1^, ^57^ a significant pathway from SVD to GM volume was also present. Although the mechanisms of brain atrophy in the setting of SVD are not fully understood, various potential explanations have been proposed, including cortical ischemia secondary to microvessel occlusions or chronic hypo‐perfusion, transneuronal degeneration secondary to axonal damage, and WM tract disruption with resulting atrophy.^57^, ^72^

SEM analysis also revealed that the effect of cardiovascular disease risk on cognition was completely mediated by SVD through pathways that both involve and are independent of brain atrophy. Our findings corroborate those of prior population‐based studies linking higher cardiovascular disease risk to worse cognitive performance.^59^ Importantly, they also provide mathematical evidence that SVD mediates this relationship.

### 4.3 Associations between individual MRI markers and cognitive performance

In supplementary analyses, basal ganglia PVS burden was associated with worse cognitive performance (global, language, and attention), whereas frontoparietal and insular PVS burden was associated with better cognitive performance (immediate memory). These findings further support the EFA results indicating that PVS located in the basal ganglia measure SVD, whereas PVS in the frontoparietal and insular regions likely measure a different construct. Similarly, previous studies have reported associations of basal ganglia PVS with increased vascular dementia risk^73^ and cognitive impairment after cerebral ischemic events,^74^ suggesting a link to cognitive decline through vascular injury‐related pathways. In contrast, studies that have focused on total PVS burden have yielded inconsistent findings, emphasizing the importance of considering PVS location when examining their relationships with cognitive outcomes. Specifically, while a risk factor‐adjusted meta‐analysis of seven longitudinal studies reported an association between PVS and impaired cognition,^75^ another meta‐analysis of five population‐based studies concluded that PVS were not associated with cognitive dysfunction.^6^

Both cross‐sectional and longitudinal associations of WMH with decreased global and domain‐specific cognitive performance have been reported.^3^, ^4^ Importantly, prior study findings also suggest a regional specificity of these associations with periventricular WMH.^12^, ^26^, ^27^ Our results replicate these findings, as WMH burden in periventricular, but not in subcortical, locations was associated with worse global, delayed memory, language, phonemic fluency, and attention cognitive performance, even after adjustment for vascular risk factors and *APOE* genotype.

While in previous studies MB have been associated with cognitive decline and incident dementia,^7^, ^8^, ^76^ we did not observe relationships between infratentorial, deep, or lobar MB presence and cognitive performance. Insufficient power of this study to detect such relationships, if they indeed exist, is a possibility. However, prior associations have been predominantly established in White participants; therefore, they might not be generalizable to the racially and ethnically diverse MESA sample. Finally, it should be noted that the absence of cross‐sectional associations in the present study does not preclude associations with cognitive decline longitudinally.

In accordance with prior reports, measures indicating worse WM microstructural integrity were associated with worse global and domain‐specific (delayed memory, executive, language, phonemic fluency, and attention) cognitive performance.^77^, ^78^

### 4.4 Limitations

This study has limitations. First, we were not able to interpret the second dimension of our two‐factor model; the nature of this construct needs to be further studied. Second, the relationships between MRI markers and cognition were examined cross‐sectionally, preventing inferences about their temporal sequence. Third, the conceptual models for the effects of age and cardiovascular disease risk on cognition represent empirical approximations of the underlying truth based on theoretical justification and available prior knowledge; alternative explanations for the observed relationships might exist. Fourth, although we considered a wide array of MRI markers, future research should also consider other SVD endophenotypes, such as lacunes, to capture other aspects of SVD. Fifth, while our sensitivity analysis (Methods 2.5.2, Supplementary Methods S3, and Results 3.4) largely shows our inferences' robustness to potential inter‐site/scanner‐related differences, further experiments are needed to assess the performance of the deep learning methods^28^, ^29^ in out‐of‐sample cohorts. In future work, we plan to examine the reliability and test–retest repeatability of these methods across different cohorts.^79^, ^80^ Lastly, despite the wide range of potential research applications of our proposed SVD construct, as a more accurate model of SVD neurobiology compared to individual MRI markers, the inclusion of DTI indices, which are not yet readily available in routine clinical settings, might make clinical practice applications more challenging. However, DTI measures will likely become more widely available in the near future, as standardized toolkits for their calculation are being developed.^81^

## 5 CONCLUSION

In summary, by utilizing cutting‐edge deep learning‐based MRI detection algorithms and latent variable analytic strategies, we integrated information from multiple SVD imaging endophenotypes and their spatial features to study the global impact of SVD on cognitive function. Our findings suggest that the cumulative impact of SVD on cognitive outcomes might be better captured by considering multiple SVD endophenotypes in strategic locations, rather than relying on individual MRI markers. Additionally, we demonstrated that SVD appears to mediate the effects of age and cardiovascular disease risk on cognition through pathways that both involve and are independent of brain atrophy. These results are of scientific interest, providing insights into the associations of SVD with, and mediating effects on, cognitive performance, while proposing analytical frameworks for modeling SVD that could enhance the understanding of its risk factors and clinical correlates. They might also be of public health relevance, highlighting the role of SVD and cardiovascular disease risk factor management in cognitive health maintenance strategies.

## CONFLICT OF INTEREST STATEMENT

The authors report no disclosures relevant to the present manuscript. Author disclosures are available in the supporting information.

## CONSENT STATEMENT

All study participants provided informed consent.

## Supporting information



Supporting Information

Supporting Information

Supporting Information

Supporting Information
