# Supplementary material for: Assessing the Global Impact of Brain Small Vessel Disease on Cognition: The Multi‐Ethnic Study of Atherosclerosis
Source: Alzheimers Dement. 2025 Jun 4;21(6):e70326. doi: 10.1002/alz.70326 (PMC12136095; doi:10.1002/alz.70326)
Supplement: Supplementary file 1 — Supporting Information [file ALZ-21-e70326-s004.docx]

| **Table S1:** Cognitive tests included in the calculation of domain-specific and global cognitive scores. | |
| --- | --- |
| *Cognitive score* | *Neuropsychological tests* |
| Immediate memory domain z-score | Craft Story Immediate, AVLT Immediate |
| Delayed memory domain z-score | Craft Story Delayed, Benson Figure Delayed, AVLT Delayed |
| Executive domain z-score | Trail Making Test Part B, Digit Span Backward |
| Language/semantic fluency domain z-score | Animals, Vegetables |
| Phonemic fluency domain z-score | Verbal Fluency: F |
| Attention/processing speed domain z-score | Trail Making Test Part A, Digit Span Forward, Digit Symbol Coding |
| Visuospatial domain z-score | Benson Figure Immediate |
| Global composite cognitive score | All normalized scores from cognitively normal participants (i.e., Craft Story Immediate, AVLT Immediate, Craft Story Delayed, Benson Figure Delayed, AVLT Delayed, Trail Making Test Part B, Digit Span Backward, Animals, Vegetables, Verbal Fluency: F, Trail Making Test Part A, Digit Span Forward, Digit Symbol Coding, and Benson Figure Immediate) were entered into a principal component analysis, and the first principal component loadings were used as weights to calculate a global composite cognitive score as a weighted sum of all individual test scores |
| Abbreviations: AVLT, Auditory-Verbal Learning Test.  *Note.* Higher global and domain-specific cognitive scores indicate better cognitive performance. | |

| **Table S2:** Participant characteristics by cognitive data availability. | | | | | |
| --- | --- | --- | --- | --- | --- |
|  | | Total (N = 1,041) | Available cognitive data | | P value |
|  |  |  | Yes (n = 892) | No (n = 149) |  |
| *Demographic and vascular risk factors* | | | | | |
| Age, mean (SD), years | | 74.1 (8.1) | 73.6 (7.9) | 77.5 (8.9) | **<0.001** ^a^ |
| Sex, n (%) | Women | 552 (53%) | 477 (53%) | 75 (50%) | 0.48 |
|  | Men | 489 (47%) | 415 (47%) | 74 (50%) |  |
| Race/ethnicity, n (%) | White | 423 (41%) | 379 (42%) | 44 (30%)^b^ | **<0.001** ^a^ |
|  | Chinese American | 155 (15%) | 119 (13%) | 36 (24%)^b^ |  |
|  | Black | 261 (25%) | 237 (27%) | 24 (16%)^b^ |  |
|  | Hispanic | 202 (19%) | 157 (18%) | 45 (30%)^b^ |  |
| Education, n (%) | Highschool or lower | 272 (26%) | 213 (24%) | 59 (40%)^b^ | **0.001** ^a^ |
|  | Less than bachelor’s degree | 313 (30%) | 272 (30%) | 41 (27%) |  |
|  | Bachelor’s degree | 212 (20%) | 187 (21%) | 25 (17%) |  |
|  | Graduate/professional school | 244 (24%) | 220 (25%) | 24 (16%)^b^ |  |
| Systolic blood pressure, mean (SD), mmHg | | 126.7 (20.6) | 126.6 (19.9) | 127.0 (24.3) | 0.84 |
|  |  | Missing (n): 2 | Missing (n): 2 | Missing (n): 0 |  |
| Use of antihypertensives, n (%) | No | 429 (41%) | 378 (43%) | 51 (34%) | 0.06 |
|  | Yes | 606 (59%) | 509 (57%) | 97 (66%) |  |
|  | Missing (n) | 6 | 5 | 1 |  |
| Diabetes mellitus, n (%) | No | 804 (78%) | 702 (79%) | 102 (70%) | **0.01** ^a^ |
|  | Yes | 225 (22%) | 182 (21%) | 43 (30%) |  |
|  | Missing (n) | 12 | 8 | 4 |  |
| Hyperlipidemia, n (%) | No | 565 (55%) | 487 (55%) | 78 (53%) | 0.62 |
|  | Yes | 470 (45%) | 400 (45%) | 70 (47%) |  |
|  | Missing (n) | 6 | 5 | 1 |  |
| Current cigarette smoking, n (%) | No | 979 (94%) | 841 (94%) | 138 (93%) | 0.62 |
|  | Yes | 61 (6%) | 51 (6%) | 10 (7%) |  |
|  | Missing (n) | 1 | 0 | 1 |  |
| Current alcohol use,  n (%) | No | 561 (54%) | 466 (52%) | 95 (64%) | **0.007** ^a^ |
|  | Yes | 479 (46%) | 426 (48%) | 53 (36%) |  |
|  | Missing (n) | 1 | 0 | 1 |  |
| Waist-to-hip ratio, mean (SD), cm/cm | | 0.94 (0.08) | 0.93 (0.08) | 0.95 (0.07) | **0.02** ^a^ |
| Total intentional exercise, median (IQR), MET/min/week | | 1,050.0  (315.0, 2,347.5) | 1,102.5  (325.0, 2,460.0) | 735.0  (131.3, 1,560.0) | **0.003** ^a^ |
|  |  | Missing (n): 3 | Missing (n): 2 | Missing (n): 1 |  |
| Framingham risk score for global cardiovascular disease, mean (SD) | | 17 (9) | 17 (9) | 20 (9) | **0.003** ^a^ |
|  |  | Missing (n): 11 | Missing (n): 8 | Missing (n): 3 |  |
| *APOE* ε4 allele carrier,  n (%) | No | 718 (72%) | 615 (73%) | 103 (72%) | 0.80 |
|  | Yes | 274 (28%) | 233 (27%) | 41 (28%) |  |
|  | Missing (n) | 49 | 44 | 5 |  |
| *Brain MRI measures of interest* | | | | | |
| Intracranial volume, mean (SD), mm^3^ | | 1,358,871 (145,134) | 1,358,887 (145,867) | 1,358,779 (141,142) | 0.99 |
| Gray matter volume, mean (SD), mm^3^ | | 596,736 (64,807) | 599,773 (64,803) | 578,556 (61,993) | **<0.001** ^a^ |
| PVS, mean (SD), count | Basal ganglia | 63.7 (16.2) | 63.3 (16.0) | 66.2 (17.5) | **0.04** ^a^ |
|  | Frontoparietal | 402.7 (155.8) | 403.9 (154.2) | 395.7 (165.7) | 0.55 |
|  | Temporal | 121 (60.1) | 122.4 (60.4) | 113.2 (57.6) | 0.08 |
|  | Insular | 5.9 (5.1) | 5.8 (5.1) | 6.0 (4.7) | 0.78 |
|  | Brainstem | 9.2 (3.7) | 9.2 (3.6) | 9.0 (4.0) | 0.52 |
|  | Thalamus | 8.8 (5.1) | 8.7 (5.1) | 9.2 (5.5) | 0.3 |
| WMH volume, median (IQR), mm^3^ | Periventricular | 2,405 (1,019, 6,391) | 2,351 (998, 6,143) | 2,881 (1,288, 7,771) | 0.07 |
|  | Subcortical | 264 (45, 1,006) | 251 (43, 946) | 281 (67, 1,716) | 0.13 |
| Presence of microbleeds,  n (%) | Infratentorial | 81 (8%) | 67 (8%) | 14 (9%) | 0.43 |
|  | Deep | 145 (14%) | 121 (14%) | 24 (16%) | 0.41 |
|  | Lobar | 245 (24%) | 216 (24%) | 29 (19%) | 0.21 |
| Mean white matter DTI measures | Fractional anisotropy, mean (SD) | 0.392 (0.025) | 0.393 (0.025) | 0.386 (0.027) | **0.002** ^a^ |
|  | Trace, mean (SD) | 0.0025 (0.0001) | 0.0025 (0.0001) | 0.0026 (0.0001) | **<0.001** ^a^ |
| Abbreviations: MET, metabolic equivalent of task; *APOE*, apolipoprotein E; PVS, perivascular spaces; WMH, white matter hyperintensities; DTI, diffusion tensor imaging.  Participant characteristics were compared using analysis of variance and Mann-Whitney-Wilcoxon tests for normally and non-normally distributed continuous variables, respectively, and Pearson’s chi-square for categorical variables.  ^a^ Significant at p ≤.05.  ^b^ Significantly different column proportion compared to participants with available cognitive data. | | | | | |

| **Table S3:** Extended analysis results. | | |
| --- | --- | --- |
| Factor loadings of the extended variables on the original factors ^a^ | | |
|  | Factor 1 | Factor 2 |
| *Perivascular spaces* | | |
| Brainstem | 0.03 | 0.29 |
| *Microbleeds* | | |
| Infratentorial | 0.31 | 0.09 |
| Deep | **0.44** ^b^ | -0.01 |
| Lobar | 0.15 | 0.05 |
| ^a^ Values represent standardized factor loadings.  ^b^ Factor loading of magnitude ≥0.32.  Note. Extended variable loadings were approximated using the Dwyer's factor extension method. | | |

| **Table S4:** Fit indices of the small vessel disease measurement model and the SEM models. | |
| --- | --- |
| *Measurement model of small vessel disease ^a^* | |
| Scaled chi-square (*χ*^2^) ^c^  Robust CFI ^c^  Robust RMSEA ^c^  SRMR | *χ*^2^ (degrees of freedom = 6, N = 892) = 8.521, p = 0.2  0.998  0.027 (90% CI, 0.000 – 0.065)  0.012 |
| *SEM model (including both measurement and structural parts) with age as the independent variable ^b^* | |
| Scaled chi-square (*χ*^2^) ^c^  Robust CFI ^c^  Robust RMSEA ^c^  SRMR | *χ*^2^ (degrees of freedom = 86, N = 892) = 367.478, p <0.001  0.928  0.069 (90% CI, 0.062 – 0.076)  0.034 |
| *SEM model (including both measurement and structural parts) with Framingham Risk Score as the independent variable ^b^* | |
| Scaled chi-square (*χ*^2^) ^c^  Robust CFI ^c^  Robust RMSEA ^c^  SRMR | *χ*^2^ (degrees of freedom = 81, N = 884) = 337.533, p <0.001  0.927  0.067 (90% CI, 0.060 – 0.074)  0.034 |
| Abbreviations: SEM, structural equation modelling; CFI, comparative fit index; RMSEA, root mean square error of approximation; SRMR, standardized root mean squared residual.  ^a^ SVD is modelled as a latent variable with perivascular spaces in the basal ganglia and thalamus, periventricular and subcortical white matter hyperintensities, and mean white matter fractional anisotropy and trace as indicators.  ^b^ Structural models include the global cognitive score as the dependent variable, and SVD and gray matter volume as mediators.  ^c^ Test statistics and fit indices that are robust to non-normality have been calculated based on the mean-and-variance corrected chi-square.^1^ | |

| **Table S5:** Alternative specifications of the models studying the mediating effects of small vessel disease in the relationships of age and cardiovascular disease risk with cognition. | | | | | | | |
| --- | --- | --- | --- | --- | --- | --- | --- |
| *Model with global cognitive score as the dependent variable, age as the independent variable, and SVD and GM volume as mediators* | | | | | | | |
| SVD modelled as ^a^ | Total effect  (95% CI) | Direct effect  (95% CI) | Indirect via SVD  (95% CI) | Indirect via GM  (95% CI) | | Total indirect effect  (95% CI) | R^2 b^ |
| Basal ganglia PVS | **-0.071 (-0.089 - -0.053)** ^d^ | **-0.025 (-0.046 - -0.002)** ^d^ | **-0.009 (-0.015 - -0.004)** ^d^ | **-0.037 (-0.053 - -0.022)** ^d^ | | **-0.046 (-0.062 - -0.031)** ^d^ | 0.217 |
| Thalamus PVS | **-0.071 (-0.089 - -0.053)** ^d^ | **-0.029 (-0.051 - -0.007)** ^d^ | **-0.003 (-0.006 - -0.0002)** ^d^ | **-0.039 (-0.054 - -0.024)** ^d^ | | **-0.042 (-0.058 - -0.026)** ^d^ | 0.211 |
| Periventricular WMH | **-0.071 (-0.089 - -0.053)** ^d^ | -0.020 (-0.043 - 0.003) | **-0.019 (-0.027 - -0.011)** ^d^ | **-0.033 (-0.047 - -0.018)** ^d^ | | **-0.051 (-0.068 - -0.035)** ^d^ | 0.222 |
| Subcortical WMH | **-0.071 (-0.089 - -0.053)** ^d^ | **-0.029 (-0.051 - -0.006)** ^d^ | **-0.005 (-0.010 - -0.001)** ^d^ | **-0.037 (-0.053 - -0.022)** ^d^ | | **-0.042 (-0.058 - -0.027)** ^d^ | 0.213 |
| WM Fractional anisotropy | **-0.071 (-0.089 - -0.053)** ^d^ | **-0.026 (-0.049 - -0.004)** ^d^ | **-0.016 (-0.025 - -0.008)** ^d^ | **-0.029 (-0.043 - -0.015)** ^d^ | | **-0.045 (-0.061 - -0.029)** ^d^ | 0.216 |
| WM Trace | **-0.071 (-0.089 - -0.053)** ^d^ | -0.016 (-0.039 - 0.008) | **-0.033 (-0.045 - -0.021)** ^d^ | **-0.023 (-0.035 - -0.010)** ^d^ | | **-0.055 (-0.073 - -0.038)** ^d^ | 0.225 |
| Latent variable ^c^ | **-0.071 (-0.089 - -0.053)** ^d^ | -0.003 (-0.028 - 0.022) | **-0.050 (-0.068 - -0.033)** ^d^ | **-0.018 (-0.030 - -0.007)** ^d^ | | **-0.068 (-0.089 - -0.049)** ^d^ | 0.239 |
| *Model with global cognitive score as the dependent variable, the Framingham Risk Score as the independent variable, and SVD and GM volume as mediators* | | | | | | | |
| SVD modelled as ^a^ | Total effect  (95% CI) | Direct effect  (95% CI) | Indirect via SVD  (95% CI) | | Indirect via GM  (95% CI) | Total indirect effect  (95% CI) | R^2 b^ |
| Basal ganglia PVS | **-0.028 (-0.044 - -0.012)** ^d^ | -0.007 (-0.023 - 0.009) | **-0.008 (-0.013 - -0.004)** ^d^ | | **-0.013 (-0.019 - -0.008)** ^d^ | **-0.021 (-0.028 - -0.014)** ^d^ | 0.206 |
| Thalamus PVS | **-0.028 (-0.044 - -0.012)** ^d^ | -0.010 (-0.026 - 0.006) | **-0.003 (-0.005 - -0.001)** ^d^ | | **-0.015 (-0.022 - -0.010)** ^d^ | **-0.018 (-0.025 - -0.012)** ^d^ | 0.199 |
| Periventricular WMH | **-0.028 (-0.044 - -0.012)** ^d^ | -0.007 (-0.023 - 0.009) | **-0.011 (-0.017 - -0.008)** ^d^ | | **-0.009 (-0.015 - -0.005)** ^d^ | **-0.021 (-0.028 - -0.015)** ^d^ | 0.212 |
| Subcortical WMH | **-0.028 (-0.044 - -0.012)** ^d^ | -0.011 (-0.027 - 0.005) | **-0.002 (-0.005 - -0.001)** ^d^ | | **-0.015 (-0.021 - -0.009)** ^d^ | **-0.017 (-0.024 - -0.011)** ^d^ | 0.201 |
| WM Fractional anisotropy | **-0.028 (-0.044 - -0.012)** ^d^ | -0.006 (-0.022 - 0.010) | **-0.015 (-0.021 - -0.010)** ^d^ | | **-0.007 (-0.012 - -0.003)** ^d^ | **-0.022 (-0.030 - -0.016)** ^d^ | 0.206 |
| WM Trace | **-0.028 (-0.044 - -0.012)** ^d^ | -0.003 (-0.019 - 0.013) | **-0.022 (-0.029 - -0.016)** ^d^ | | **-0.004 (-0.007 - -0.001)** ^d^ | **-0.025 (-0.033 - -0.018)** ^d^ | 0.217 |
| Latent variable ^c^ | **-0.028 (-0.044 - -0.012)** ^d^ | 0.001 (-0.016 - 0.018) | **-0.027 (-0.036 - -0.020)** ^d^ | | -0.002 (-0.005 - 0.000) | **-0.029 (-0.038 - -0.021)** ^d^ | 0.229 |
| Abbreviations: SVD, small vessel disease; GM, gray matter; PVS, perivascular spaces; WMH white matter hyperintensities; WM, white matter.  ^a^ SVD is modelled either as a latent variable, or as an observed variable using each of the individual MRI markers assumed to measure SVD.  ^b^ Proportion of variation in global cognitive score explained by the model.  ^c^ SVD modelled as a latent variable measured by PVS in the basal ganglia and thalamus, periventricular and subcortical WMH, and WM FA and TR.  ^d^ Significant at p ≤.05. | | | | | | | |

| **Table S6:** Associations of MRI markers of small vessel disease with cognitive performance (Model 1)^a^. | | | | | | | | | | | | | | |
| --- | --- | --- | --- | --- | --- | --- | --- | --- | --- | --- | --- | --- | --- | --- |
| Cognitive Domain | | Perivascular spaces | | | | | | WMH | | Microbleeds | | | Mean WM DTI measures | |
|  |  | Basal ganglia | Fronto-parietal | Temporal | Insular | Brainstem | Thalamus | Periventri-cular, x^0.075^ | Subcortical, x^0.2^ | Infratentorial | Deep | Lobar | Fractional  anisotropy | Trace |
| Global | β | **-0.243** ^b^ | 0.129 | 0.136 | 0.104 | 0.036 | -0.12 | **-0.363** ^b^ | **-0.186** ^b^ | -0.098 | -0.263 | -0.089 | **0.304** ^b^ | **-0.441** ^b^ |
|  | 95%  CI | **-0.381,**  **-0.104** | -0.017,  0.275 | -0.006,  0.278 | -0.028,  0.237 | -0.100,  0.172 | -0.256,  0.016 | **-0.512,**  **-0.213** | **-0.325,**  **-0.047** | -0.598,  0.403 | -0.651,  0.125 | -0.396,  0.218 | **0.156,**  **0.452** | **-0.598,**  **-0.283** |
| Immediate  Memory | β | -0.016 | **0.086** ^b^ | 0.077 | **0.085** ^b^ | 0.009 | -0.03 | -0.051 | -0.044 | 0.097 | -0.072 | 0.019 | 0.047 | -0.058 |
|  | 95%  CI | -0.076,  0.043 | **0.024,**  **0.148** | 0.017,  0.137 | **0.029,**  **0.141** | -0.049,  0.067 | -0.088,  0.028 | -0.116,  0.013 | -0.103,  0.015 | -0.115,  0.310 | -0.237,  0.093 | -0.112,  0.150 | -0.016,  0.111 | -0.126,  0.010 |
| Delayed  Memory | β | -0.063 | 0.056 | 0.04 | 0.059 | -0.011 | -0.03 | **-0.108** ^b^ | -0.063 | 0.021 | -0.118 | 0.024 | **0.104** ^b^ | **-0.148** ^b^ |
|  | 95%  CI | -0.122,  -0.005 | -0.005,  0.117 | -0.020,  0.100 | 0.003,  0.114 | -0.068,  0.046 | -0.087,  0.027 | **-0.171,**  **-0.045** | -0.122,  -0.005 | -0.189,  0.231 | -0.281,  0.045 | -0.104,  0.153 | **0.042,**  **0.166** | **-0.215,**  **-0.081** |
| Executive | β | -0.051 | 0.063 | 0.068 | 0.008 | 0.012 | -0.023 | **-0.1** ^b^ | -0.069 | 0.027 | -0.005 | -0.121 | 0.046 | **-0.137** ^b^ |
|  | 95%  CI | -0.117,  0.015 | -0.007,  0.133 | 0.001,  0.136 | -0.055,  0.071 | -0.053,  0.077 | -0.088,  0.042 | **-0.171,**  **-0.028** | -0.135,  -0.002 | -0.211,  0.265 | -0.189,  0.180 | -0.266,  0.025 | -0.025,  0.117 | **-0.213,**  **-0.062** |
| Language/  semantic  fluency | β | **-0.098** ^b^ | 0.003 | 0.029 | 0.023 | 0.019 | -0.056 | **-0.119** ^b^ | -0.049 | -0.194 | -0.046 | -0.032 | **0.141** ^b^ | **-0.165** ^b^ |
|  | 95%  CI | **-0.160,**  **-0.036** | -0.062,  0.068 | -0.034,  0.092 | -0.036,  0.082 | -0.041,  0.079 | -0.116,  0.004 | **-0.185,**  **-0.052** | -0.111,  0.013 | -0.415,  0.027 | -0.218,  0.126 | -0.168,  0.104 | **0.075,**  **0.206** | **-0.236,**  **-0.094** |
| Phonemic fluency | β | -0.054 | 0.037 | 0.059 | 0.063 | 0.033 | -0.016 | **-0.118** ^b^ | -0.076 | 0.003 | -0.165 | -0.151 | 0.086 | **-0.125** ^b^ |
|  | 95%  CI | -0.126,  0.018 | -0.037,  0.111 | -0.012,  0.131 | -0.005,  0.131 | -0.037,  0.103 | -0.086,  0.055 | **-0.194,**  **-0.041** | -0.147,  -0.006 | -0.256,  0.262 | -0.358,  0.029 | -0.305,  0.003 | 0.010,  0.162 | **-0.209,**  **-0.041** |
| Attention/ processing  Speed | β | **-0.115** ^b^ | 0.004 | 0.006 | -0.02 | 0.021 | -0.053 | **-0.151** ^b^ | -0.063 | -0.041 | -0.061 | 0.021 | **0.119** ^b^ | **-0.164** ^b^ |
|  | 95%  CI | **-0.174,**  **-0.056** | -0.058,  0.067 | -0.054,  0.067 | -0.077,  0.036 | -0.037,  0.079 | -0.111,  0.005 | **-0.215,**  **-0.088** | -0.122,  -0.004 | -0.254,  0.172 | -0.226,  0.105 | -0.110,  0.151 | **0.056,**  **0.182** | **-0.232,**  **-0.097** |
| Visuo-  Spatial | β | -0.081 | 0.074 | 0.033 | 0.052 | -0.032 | 0.008 | -0.066 | 0.046 | -0.172 | 0.046 | 0.075 | 0.108 | -0.11 |
|  | 95%  CI | -0.188,  0.026 | -0.033,  0.182 | -0.071,  0.136 | -0.046,  0.149 | -0.134,  0.070 | -0.092,  0.107 | -0.182,  0.051 | -0.060,  0.151 | -0.556,  0.212 | -0.237,  0.329 | -0.158,  0.308 | -0.005,  0.221 | -0.230,  0.011 |
| Abbreviations: WMH, white matter hyperintensities; WM, white matter; DTI, diffusion tensor imaging.  ^a^ Values represent beta coefficients from generalized linear models with cognitive scores as the outcomes and the respective small vessel disease marker as the main predictor. Models were adjusted for age, sex, race/ethnicity, education, study site, total intracranial volume, language of cognitive testing, interval between MRI scan and cognitive testing, and order of MRI scan and cognitive testing completion.  ^b^ Significant after false-discovery rate control at <5% within each cognitive domain using the Benjamini-Hochberg procedure.  Regional WMH volumes have undergone Tukey's "ladder of powers" transformation. | | | | | | | | | | | | | | |

| **Table S7:** Associations of MRI markers of small vessel disease with cognitive performance after adjustment for vascular risk factors (Model 2)^a^. | | | | | | | | | | | | | | |
| --- | --- | --- | --- | --- | --- | --- | --- | --- | --- | --- | --- | --- | --- | --- |
| Cognitive Domain | | Perivascular spaces | | | | | | WMH | | Microbleeds | | | Mean WM DTI measures | |
|  |  | Basal ganglia | Fronto-parietal | Temporal | Insular | Brainstem | Thalamus | Periventri-cular, x^0.075^ | Subcortical, x^0.2^ | Infratentorial | Deep | Lobar | Fractional  anisotropy | Trace |
| Global | β | **-0.213** ^b^ | 0.1 | 0.107 | 0.099 | -0.009 | -0.127 | **-0.362** ^b^ | **-0.175** ^b^ | -0.09 | -0.181 | -0.056 | **0.296** ^b^ | **-0.422** ^b^ |
|  | 95%  CI | **-0.353,**  **-0.072** | -0.046,  0.246 | -0.035,  0.249 | -0.033,  0.230 | -0.146,  0.128 | -0.264,  0.010 | **-0.514,**  **-0.211** | **-0.315,**  **-0.035** | -0.592,  0.412 | -0.576,  0.215 | -0.362,  0.251 | **0.144,**  **0.448** | **-0.585,**  **-0.258** |
| Immediate  memory | β | -0.009 | 0.067 | 0.054 | 0.074 | -0.004 | -0.039 | -0.063 | -0.045 | 0.106 | -0.064 | 0.021 | 0.053 | -0.059 |
|  | 95%  CI | -0.069,  0.052 | 0.005,  0.129 | -0.006,  0.115 | 0.018,  0.130 | -0.062,  0.054 | -0.097,  0.019 | -0.128,  0.002 | -0.104,  0.015 | -0.106,  0.319 | -0.231,  0.104 | -0.110,  0.151 | -0.012,  0.118 | -0.130,  0.011 |
| Delayed  memory | β | -0.052 | 0.048 | 0.032 | 0.055 | -0.029 | -0.033 | **-0.109** ^b^ | -0.062 | 0.021 | -0.091 | 0.032 | **0.101** ^b^ | **-0.139** ^b^ |
|  | 95%  CI | -0.111,  0.008 | -0.013,  0.110 | -0.028,  0.091 | -0.001,  0.110 | -0.087,  0.028 | -0.091,  0.024 | **-0.173,**  **-0.045** | -0.121,  -0.004 | -0.190,  0.232 | -0.257,  0.075 | -0.097,  0.161 | **0.037,**  **0.165** | **-0.208,**  **-0.070** |
| Executive | β | -0.047 | 0.058 | 0.065 | 0.015 | 0.002 | -0.022 | -0.097 | -0.063 | 0.042 | 0.03 | -0.104 | 0.032 | -0.116 |
|  | 95%  CI | -0.115,  0.021 | -0.012,  0.128 | -0.004,  0.133 | -0.049,  0.078 | -0.064,  0.068 | -0.088,  0.044 | -0.170,  -0.023 | -0.130,  0.004 | -0.200,  0.283 | -0.161,  0.220 | -0.252,  0.043 | -0.041,  0.106 | -0.196,  -0.036 |
| Language/  semantic  fluency | β | **-0.095** ^b^ | -0.005 | 0.023 | 0.024 | 0.004 | -0.06 | **-0.119** ^b^ | -0.05 | -0.216 | -0.028 | -0.03 | **0.144** ^b^ | **-0.165** ^b^ |
|  | 95%  CI | **-0.158,**  **-0.031** | -0.071,  0.061 | -0.041,  0.087 | -0.035,  0.084 | -0.058,  0.065 | -0.121,  0.002 | **-0.188,**  **-0.050** | -0.113,  0.013 | -0.441,  0.009 | -0.205,  0.150 | -0.168,  0.108 | **0.076,**  **0.212** | **-0.239,**  **-0.091** |
| Phonemic fluency | β | -0.042 | 0.028 | 0.053 | 0.059 | 0.025 | -0.011 | **-0.118** ^b^ | -0.07 | -0.021 | -0.172 | -0.139 | 0.081 | -0.116 |
|  | 95%  CI | -0.116,  0.032 | -0.047,  0.103 | -0.020,  0.126 | -0.010,  0.127 | -0.047,  0.097 | -0.083,  0.061 | **-0.196,**  **-0.039** | -0.142,  0.002 | -0.287,  0.244 | -0.373,  0.028 | -0.296,  0.018 | 0.002,  0.160 | -0.203,  -0.028 |
| Attention/ processing  speed | β | **-0.111** ^b^ | -0.002 | 0.000 | -0.018 | 0.013 | -0.051 | **-0.151** ^b^ | -0.06 | -0.042 | -0.033 | 0.036 | **0.107** ^b^ | **-0.141** ^b^ |
|  | 95%  CI | **-0.172,**  **-0.051** | -0.065,  0.061 | -0.062,  0.061 | -0.075,  0.038 | -0.046,  0.072 | -0.110,  0.008 | **-0.217,**  **-0.086** | -0.120,  0.001 | -0.258,  0.175 | -0.204,  0.137 | -0.096,  0.168 | **0.041,**  **0.173** | **-0.212,**  **-0.071** |
| Visuo-  spatial | β | -0.065 | 0.052 | 0.008 | 0.035 | -0.069 | 0.017 | -0.058 | 0.053 | -0.164 | 0.096 | 0.127 | 0.087 | -0.087 |
|  | 95%  CI | -0.174,  0.045 | -0.056,  0.159 | -0.096,  0.113 | -0.062,  0.133 | -0.173,  0.034 | -0.084,  0.118 | -0.177,  0.061 | -0.053,  0.159 | -0.555,  0.226 | -0.195,  0.387 | -0.107,  0.361 | -0.030,  0.204 | -0.211,  0.037 |
| Abbreviations: WMH, white matter hyperintensities; WM, white matter; DTI, diffusion tensor imaging.  ^a^ Values represent beta coefficients from generalized linear models with cognitive scores as the outcomes and the respective small vessel disease marker as the main predictor. Models were adjusted for age, sex, race/ethnicity, education, study site, total intracranial volume, language of cognitive testing, interval between MRI scan and cognitive testing, order of MRI scan and cognitive testing completion, and vascular risk factors (systolic blood pressure, use of antihypertensive medications, diabetes, hyperlipidemia, current smoking, current alcohol consumption, waist-to-hip ratio, and intentional physical activity).  ^b^ Significant after false-discovery rate control at <5% within each cognitive domain using the Benjamini-Hochberg procedure.  Regional WMH volumes have undergone Tukey's "ladder of powers" transformation. | | | | | | | | | | | | | | |

| **Table S8:** Associations of MRI markers of small vessel disease with cognitive performance after adjustment for vascular risk factors and *APOE* genotype (Model 3)^a^. | | | | | | | | | | | | | | |
| --- | --- | --- | --- | --- | --- | --- | --- | --- | --- | --- | --- | --- | --- | --- |
| Cognitive Domain | | Perivascular spaces | | | | | | WMH | | Microbleeds | | | Mean WM DTI measures | |
|  |  | Basal ganglia | Fronto-parietal | Temporal | Insular | Brainstem | Thalamus | Periventri-cular, x^0.075^ | Subcortical, x^0.2^ | Infratentorial | Deep | Lobar | Fractional  anisotropy | Trace |
| Global | β | **-0.206** ^b^ | 0.102 | 0.111 | 0.073 | 0.006 | -0.126 | **-0.362** ^b^ | -0.168 | -0.004 | -0.203 | -0.012 | **0.282** ^b^ | **-0.408** ^b^ |
|  | 95%  CI | **-0.350,**  **-0.062** | -0.046,  0.251 | -0.034,  0.255 | -0.061,  0.207 | -0.137,  0.149 | -0.268,  0.016 | **-0.519,**  **-0.205** | -0.313,  -0.023 | -0.522,  0.513 | -0.608,  0.201 | -0.328,  0.304 | **0.125,**  **0.438** | **-0.576,**  **-0.239** |
| Immediate  memory | β | -0.009 | 0.068 | 0.056 | 0.063 | -0.002 | -0.039 | -0.061 | -0.04 | 0.167 | -0.047 | 0.045 | 0.042 | -0.049 |
|  | 95%  CI | -0.071,  0.053 | 0.005,  0.131 | -0.006,  0.117 | 0.006,  0.120 | -0.063,  0.059 | -0.100,  0.021 | -0.128,  0.007 | -0.101,  0.022 | -0.052,  0.387 | -0.220,  0.125 | -0.090,  0.180 | -0.025,  0.108 | -0.122,  0.023 |
| Delayed  memory | β | -0.049 | 0.048 | 0.029 | 0.044 | -0.022 | -0.039 | **-0.103** ^b^ | -0.057 | 0.035 | -0.117 | 0.024 | **0.097** ^b^ | **-0.132** ^b^ |
|  | 95%  CI | -0.109,  0.012 | -0.014,  0.110 | -0.032,  0.089 | -0.012,  0.100 | -0.082,  0.037 | -0.099,  0.020 | **-0.170,**  **-0.037** | -0.118,  0.004 | -0.181,  0.251 | -0.286,  0.052 | -0.108,  0.156 | **0.032,**  **0.163** | **-0.202,**  **-0.061** |
| Executive | β | -0.035 | 0.067 | 0.077 | 0.009 | 0.013 | -0.016 | -0.096 | -0.066 | 0.092 | 0.03 | -0.077 | 0.027 | -0.109 |
|  | 95%  CI | -0.105,  0.034 | -0.005,  0.138 | 0.007,  0.146 | -0.055,  0.074 | -0.056,  0.082 | -0.085,  0.052 | -0.172,  -0.019 | -0.136,  0.003 | -0.157,  0.340 | -0.165,  0.224 | -0.229,  0.075 | -0.049,  0.102 | -0.191,  -0.027 |
| Language/  semantic  fluency | β | **-0.096** ^b^ | -0.011 | 0.016 | 0.015 | 0.009 | -0.055 | **-0.112** ^b^ | -0.04 | -0.184 | -0.036 | -0.034 | **0.151** ^b^ | **-0.166** ^b^ |
|  | 95%  CI | **-0.161,**  **-0.031** | -0.078,  0.056 | -0.049,  0.081 | -0.046,  0.075 | -0.055,  0.074 | -0.118,  0.009 | **-0.183,**  **-0.041** | -0.106,  0.025 | -0.416,  0.048 | -0.218,  0.146 | -0.176,  0.109 | **0.082,**  **0.221** | **-0.242,**  **-0.090** |
| Phonemic fluency | β | -0.048 | 0.032 | 0.055 | 0.06 | 0.025 | -0.02 | **-0.124** ^b^ | -0.069 | -0.06 | -0.178 | -0.119 | 0.091 | **-0.133** ^b^ |
|  | 95%  CI | -0.125,  0.028 | -0.045,  0.108 | -0.019,  0.130 | -0.011,  0.130 | -0.050,  0.099 | -0.095,  0.055 | **-0.205,**  **-0.043** | -0.143,  0.006 | -0.335,  0.215 | -0.383,  0.027 | -0.282,  0.044 | 0.010,  0.172 | **-0.222,**  **-0.043** |
| Attention/ processing  speed | β | **-0.108** ^b^ | 0.001 | 0.005 | -0.021 | 0.01 | -0.047 | **-0.157** ^b^ | -0.061 | -0.037 | -0.06 | 0.056 | **0.096** ^b^ | **-0.131** ^b^ |
|  | 95%  CI | **-0.170,**  **-0.047** | -0.063,  0.065 | -0.057,  0.067 | -0.078,  0.037 | -0.051,  0.072 | -0.107,  0.014 | **-0.224,**  **-0.089** | -0.124,  0.001 | -0.259,  0.185 | -0.233,  0.114 | -0.080,  0.192 | **0.029,**  **0.163** | **-0.204,**  **-0.058** |
| Visuo-  spatial | β | -0.067 | 0.056 | 0.016 | 0.033 | -0.044 | 0.022 | -0.084 | 0.03 | -0.232 | 0.12 | 0.109 | 0.099 | -0.123 |
|  | 95%  CI | -0.179,  0.045 | -0.055,  0.166 | -0.091,  0.123 | -0.068,  0.134 | -0.153,  0.065 | -0.084,  0.127 | -0.208,  0.040 | -0.081,  0.142 | -0.633,  0.168 | -0.180,  0.420 | -0.135,  0.352 | -0.022,  0.219 | -0.252,  0.006 |
| Abbreviations: WMH, white matter hyperintensities; WM, white matter; DTI, diffusion tensor imaging.  ^a^ Values represent beta coefficients from generalized linear models with cognitive scores as the outcomes and the respective small vessel disease marker as the main predictor. Models were adjusted for age, sex, race/ethnicity, education, study site, total intracranial volume, language of cognitive testing, interval between MRI scan and cognitive testing, order of MRI scan and cognitive testing completion, vascular risk factors (systolic blood pressure, use of antihypertensive medications, diabetes, hyperlipidemia, current smoking, current alcohol consumption, waist-to-hip ratio, and intentional physical activity), and *APOE* ε4 carrier status.  ^b^ Significant after false-discovery rate control at <5% within each cognitive domain using the Benjamini-Hochberg procedure.  Regional WMH volumes have undergone Tukey's "ladder of powers" transformation. | | | | | | | | | | | | | | |

# References

1. Asparouhov T, Muthén B. Simple Second Order Chi-Square Correction. *Mplus Technical Appendix.* 2010.
