# Supplementary material for: Assessing the Global Impact of Brain Small Vessel Disease on Cognition: The Multi‐Ethnic Study of Atherosclerosis
Source: Alzheimers Dement. 2025 Jun 4;21(6):e70326. doi: 10.1002/alz.70326 (PMC12136095; doi:10.1002/alz.70326)

**Figure S1:** Flowchart of analytic sample. MESA, Multi-Ethnic Study of Atherosclerosis; SVD, small vessel disease.

**1,062** MESA Atrial Fibrillation ancillary study participants with available brain MRI scans

**10** participants with MRI scans of poor quality due to significant distortion and/or motion artifact

**11** participants with incomplete data on SVD markers

**76** participants with available neuropsychological evaluation from Epigenetics of Cognitive Decline ancillary study

**892** participants included in the analysis

**1,041** participants with complete data on SVD markers

**149** participants with no available neuropsychological evaluation data

**701** participants with available neuropsychological evaluation from MIND ancillary study

**115** participants with available neuropsychological evaluation from MESA Memory ancillary study

**Figure S2:** The very simple structure (VSS) criterion compares solutions for various levels of item complexity and various numbers of factors. Complexity 1 solutions achieve their maxima at two factors (a). The minimum average partial (MAP) algorithm conducts a complete principal components analysis and computes a partial correlation matrix after extracting each principal component. The average squared coefficient in the off-diagonals of the resulting partial correlation matrix is then calculated. This average is at a minimum when the best number of components has been extracted. This criterion achieves its minimum after extraction of two components (b).

a)
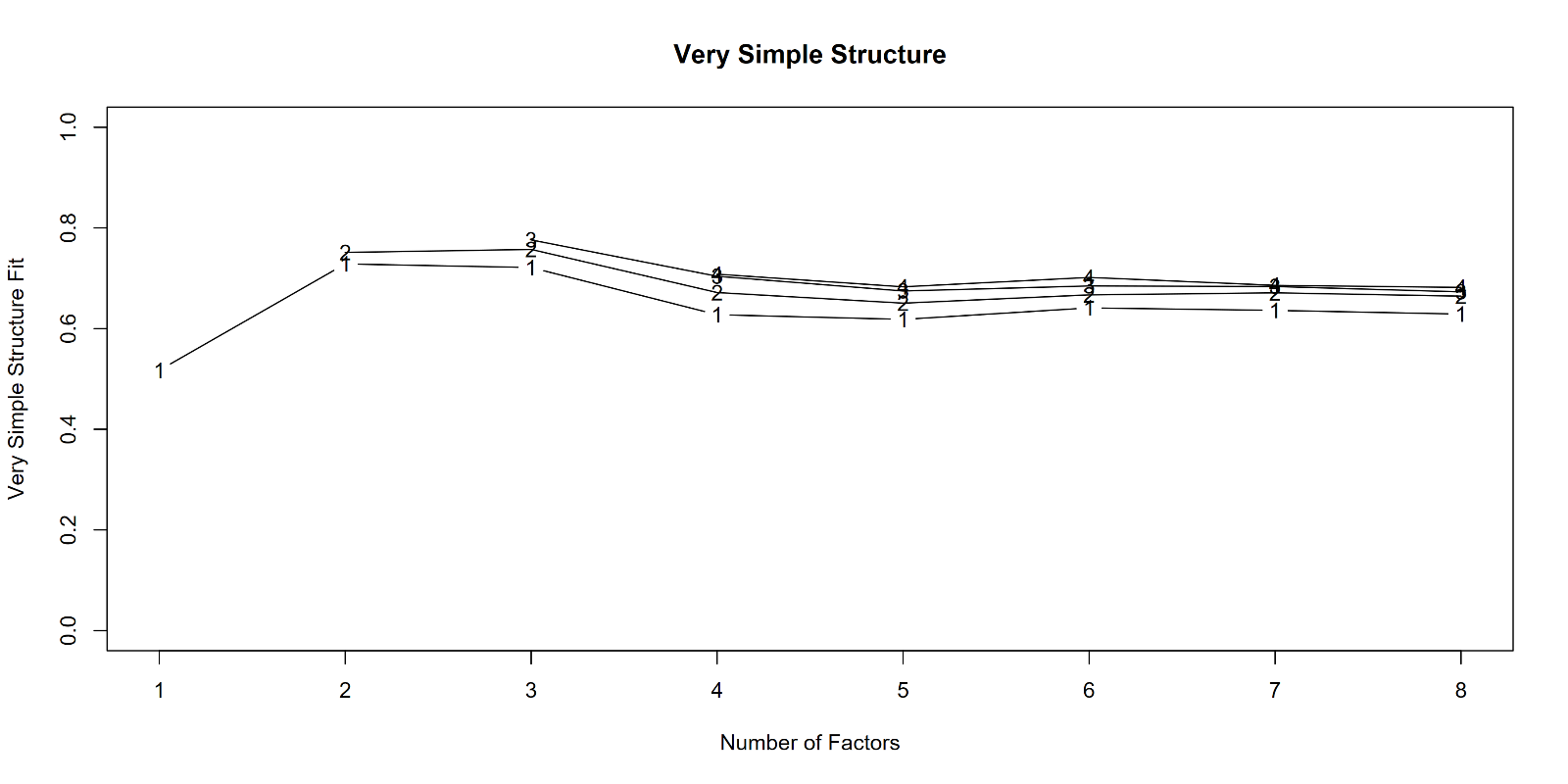


b)
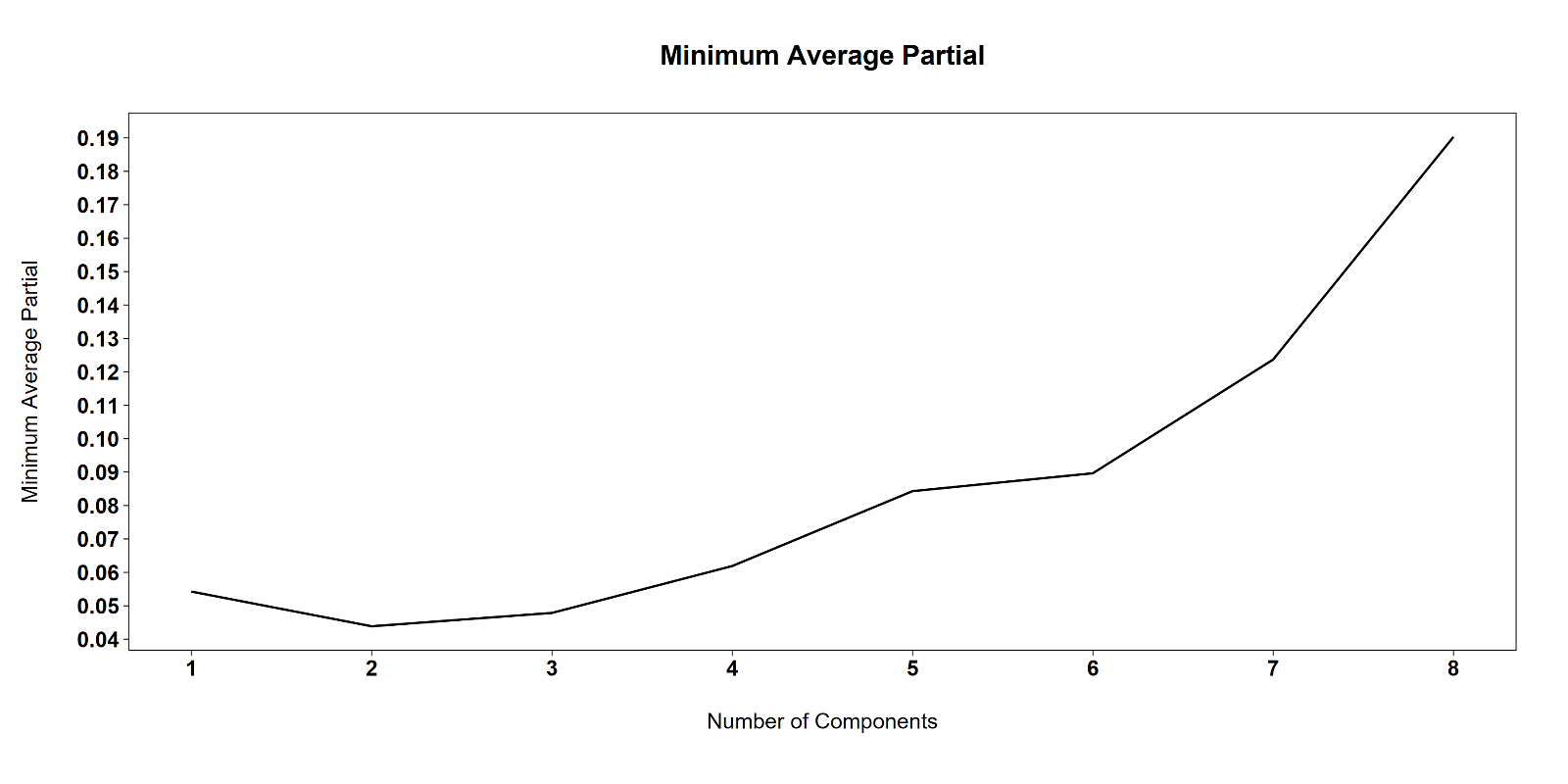

Supplement: Supplementary file 2 — Supporting Information [file ALZ-21-e70326-s003.docx]
