# Supplementary material for: Assessing the Global Impact of Brain Small Vessel Disease on Cognition: The Multi‐Ethnic Study of Atherosclerosis
Source: Alzheimers Dement. 2025 Jun 4;21(6):e70326. doi: 10.1002/alz.70326 (PMC12136095; doi:10.1002/alz.70326)
Supplement: Supplementary file 3 — Supporting Information [file ALZ-21-e70326-s002.docx]

# Methods S1: Brain MRI acquisition and quality assurance

Brain MRI scans were acquired at six different study sites (Columbia University, New York, New York; Johns Hopkins University, Baltimore, Maryland; Northwestern University, Chicago, Illinois; University of California Los Angeles, Los Angeles, California; University of Minnesota, Minneapolis, Minnesota; and Wake Forest University, Winston-Salem, North Carolina) on two 3‐Tesla (3T) Siemens scanners: Prisma VE11C (University of California Los Angeles, Columbia University, John Hopkins University, Northwestern University, University of Minnesota) and Skyra VD11B (University of California Los Angeles, Wake Forest University).^1,2^

Each MRI site followed standard quality assurance protocols using phantoms developed for the Alzheimer's Disease Neuroimaging Initiative (ADNI) and the Functional Bioinformatics Research Network (FBIRN), as previously described.^1^ The following established quality assurance acceptance thresholds from phantom scans were used: Signal-to-Noise (SNR) >300 and maximum distortion >1mm for ADNI measurements; Signal-to-Fluctuation Noise Ratio (SfNR) >220 and Radius DeCorrelation (RDC) >3.1 for FBIRN measurements. Scanner performance was met for all MRI sites prior to study start-up and was monitored throughout the study with all scanners showing stability of phantom measurements.^1^

# Methods S2: Deep learning model training

The deep learning model for the detection of perivascular spaces was trained using 21 randomly selected MESA participant scans from all sites and scanner models. The training sample included both women (n = 11) and men (n = 10), with ages ranging from 64 to 94 years (mean age: 78.7 years).^3^

The deep learning model for the detection of cerebral microbleeds was trained using 24 randomly selected MESA participant scans from all sites, except the University of Minnesota, and all scanner models. The training sample included both women (n = 11) and men (n = 13), with ages ranging from 65 to 94 years (mean age: 76.2 years).^4^

Detection results were thoroughly assessed using leave-one-out cross-validation and standard performance metrics to ensure accurate predictions, as previously described.^3,4^

# Methods S3: Metric invariance and inter-site/scanner reliability of the MRI indicators

Each MRI indicator can be expressed as a function of the small vessel disease (SVD) latent construct and measurement error.

Mathematically:

$X=\lambda F+ \varepsilon$ (1)

Where,

X denotes the MRI indicator

F denotes the latent SVD factor

λ denotes the MRI indicator loading on the latent SVD factor

ε denotes the measurement error

From equation (1), we can obtain the following variance decomposition for each MRI indicator:

$Var(X)=\lambda^{2} Var(F)+ Var(\varepsilon)$ (2)

Where,

Var(X) denotes the MRI indicator variance

Var(F) denotes the latent SVD factor variance

$Var(\varepsilon_{\iota})$ denotes the residual variance

Then, from equation (2), we can compute the reliability of each MRI indicator, which is defined as the proportion of the total MRI indicator variance that is not error variance^6^:

$r_{X}=\frac{\lambda^{2} Var(F)}{\lambda^{2} Var(F)+ Var(\varepsilon)}$ (3)

We first fitted a multi-group confirmatory factor analysis (MGCFA) model assuming only configural invariance (i.e., same SVD factor structure) across study sites.

For the configural invariance model, equations (1), (2), and (3) become:

$X_{i}=\lambda_{i} F_{i}+ \varepsilon_{i}$ (4)

$Var\left( X_{i} \right)=\lambda_{i}^{2} Var(F_{i})+ Var(\varepsilon_{i})$ (5)

$r_{X_{i}}=\frac{\lambda_{i}^{2} Var(F_{i})}{\lambda_{i}^{2} Var(F_{i})+ Var(\varepsilon_{i})}$ (6)

Where,

i denotes the study site

Subsequently, we proceeded to test metric invariance by imposing equality constraints on the MRI indicator loadings across the different study sites, and compared the configural invariance model with the metric invariance model using a likelihood ratio test with a scaled chi-square difference test statistic.^7^

Finally, we further constrained the residual variance of each MRI indicator to be the same across sites, and also the SVD latent factor variance to be equal across sites, essentially constraining all left-sided terms of equation (6) to be the same across sites – thereby imposing equal MRI indicator reliability across sites/scanners; we then compared this model with the metric invariance model using a likelihood ratio test with a scaled chi-square difference test statistic.^7^

# References

1. Austin TR, Nasrallah IM, Erus G, et al. Association of Brain Volumes and White Matter Injury With Race, Ethnicity, and Cardiovascular Risk Factors: The Multi-Ethnic Study of Atherosclerosis. *J Am Heart Assoc.* 2022;11(7):e023159.

2. Heckbert SR, Jensen PN, Erus G, et al. Heart rate fragmentation and brain MRI markers of small vessel disease in MESA. *Alzheimer's & Dementia.* 2024;20(2):1397-1405.

3. Rashid T, Liu H, Ware JB, et al. Deep learning based detection of enlarged perivascular spaces on brain MRI. *Neuroimage: Reports.* 2023;3(1):100162.

4. Rashid T, Abdulkadir A, Nasrallah IM, et al. DEEPMIR: a deep neural network for differential detection of cerebral microbleeds and iron deposits in MRI. *Sci Rep.* 2021;11(1):14124.
